# The different paradigms of NK cell death in patients with severe trauma

**DOI:** 10.1038/s41419-024-06992-0

**Published:** 2024-08-21

**Authors:** Te-Ding Chang, Deng Chen, Jia-Liu Luo, Yu-Man Wang, Cong Zhang, Shun-Yao Chen, Zhi-Qiang Lin, Pei-Dong Zhang, Ting-Xuan Tang, Hui Li, Li-Ming Dong, Ning Wu, Zhao-Hui Tang

**Affiliations:** 1 Division of Trauma Surgery, Emergency Surgery & Surgical Critical, Tongji Trauma Center, Tongji Hospital, Tongji Medical College, Huazhong University of Science and Technology, Wuhan, China; 2 Department of Emergency and Critical Care Medicine, Tongji Hospital, Tongji Medical College, Huazhong University of Science and Technology, Wuhan, China; 3 Department of Immunology, School of Basic Medicine, Tongji Medical College, Huazhong University of Science and Technology, Wuhan, China; 4 Department of Orthopedics, Tongji Hospital, Tongji Medical College, Huazhong University of Science and Technology, Wuhan, China; 5 Department of Immunology, School of Basic Medical Sciences, Anhui Medical University, Hefei, China

**Keywords:** Trauma, Innate immunity

## Abstract

Lymphocyte decline, particularly the depletion of NK cells, is a prominent feature of immunosuppression following severe tissue injury, heightening the susceptibility of severe trauma patients to life-threatening infections. Previous research indicates that the reduction in the number of NK cells is closely associated with the process of cell death. Nonetheless, the precise mechanism of NK cell death remains unknown. Here, we discovered that following severe traumatic injury, NK cells undergo several cell death pathways, dominated by apoptosis and pyroptosis with coexistence of necrotic cell death, immunogenic cell death, ferroptosis, and autophagy. These NK cells with different paradigms of death have diverse cytokine expression profiles and diverse interactions with other immune cells. Further exploration revealed that hypoxia was strongly associated with this diverse paradigm of NK cell death. Detailed investigation of paradigms of cell death may help to enhance comprehension of lymphopenia post-severe trauma, to develop new strategy in preventing immunosuppression, and then to improve outcome for severe trauma population.

## Introduction

Severe trauma represents around 10% of global mortality, primarily because of fatal damage and intractable complications [1]. The immediate cause of death following severe trauma is hemorrhage and devastating head injury, whereas the majority of delayed deaths due to complex complications [2–4]. Despite substantial improvements in resuscitation strategies to improve early survival, expediting recovery still remains a challenge due to high incidence of delayed deaths [1]. Intractable complications such as sepsis and multiple organ dysfunction syndrome [5] are the major contributing factors that are frequently attributed to trauma-hemorrhage-induced immunosuppression [6–8]. Significant lymphopenia is a prominent feature of immunosuppression in severe trauma patients [7–9]. There is a growing body of literature demonstrating lymphopenia after severe trauma has been associated with MODS and a rise in mortality rate, as well as preventing lymphopenia contributed to shorten hospital stay and to alleviate the morbidity in severe trauma patients [6–9].

This trauma-induced lymphopenia was comprehensive with reduced numbers of T cells, Natural killer (NK) cells, Natural killer T (NKT) cells and B cells [6, 10]. T cells depletion has been extensively investigated and discussed, but NK cell depletion has received considerably less attention in spite of NK cells identified as the most sharply reduced cell lymphocyte subsets after trauma [6, 10]. NK cells are essential innate immune cells and account for 10–15% of the circulating lymphocytes in humans [11]. Unlike conventional lymphocytes, NK cells with innate-like properties can be directly activated by danger-associated-molecular patterns (DAMPs) to generate acute inflammation during the hyperacute immune response to trauma, <2 h following injury [12]. So, NK cells play a crucial role in the early immunological events after the initial traumatic insult.

Profound reduction in NK cells in PBMC is frequently attributed to cell death or the migration of a circulating NK cell into target tissue [12]. Mature NK cells circulate mainly in the peripheral blood, but are also present in several lymphoid and nonlymphoid organs such as liver, spleen, lymph nodes, tonsils, lungs, intestine, and uterus [11]. Recent studied revealed no evidence of NK cells migration to potential destinations such as wounded tissue, lung, spleen and lymph nodes using the mice model of tissue trauma or/and hemorrhagic shock [12, 13].

Indeed, it is now well established that regulated cell death (RCD) plays a vital role in immune homeostasis maintenance and diseases development, and RCD is a major cause for the dramatic loss of circulating NK cells after severe trauma [14]. Based on different morphological, biochemical, immunological, and genetic characteristics, RCD is subdivided into apoptotic and non-apoptotic categories. Non-apoptotic RCD can be subdivided into autophagy, ferroptosis, pyroptosis, necroptosis, and so on [15]. In previous studies, lymphopenia is frequently attributed to cells apoptosis [6, 8]. However, recent study indicated that widespread cells apoptosis cannot be entirely responsible for loss of NK cells observed in severe trauma patients [12]. Merely inhibiting apoptosis fails to completely reverse persistent NK cell reduction during severe trauma. It has become obvious that trauma stress can lead to different types of cell death, the concept of apoptosis is not adequate to describe all forms of cell death after severe traumatic injury.

At present, little is known concerning the different forms of NK cell death after severe trauma, especially in non-apoptosis cell death. To address this issue, this study observed the different paradigms of NK cell death in patients with severe trauma. In particular, twelve cell death signaling pathways were simultaneously detected and analyzed, which helps to understand the panoramic molecular mechanisms of NK cell death induced by severe trauma. Our study revealed the distribution of each subtype of RCD in NK cells involving in trauma-induced lymphopenia. Detailed investigation of paradigms of cell death may help to develop new strategy in preventing immunosuppression and improving outcome for severe trauma population.

## Results

### Result 1. Patient clinical characteristics

From January 2021 until February 2022, we monitored the immune status of 18 patients who suffered from severe trauma while receiving treatment in the Trauma Intensive Care Unit (TICU) at the Tongji Trauma Center of Tongji Hospital. All 18 patients had an Injury Severity Score (ISS) greater than 16, with an average ISS of 21.3 ± 1.62. Upon admission, all patients were conscious no brain damage and had a Glasgow Coma Score (GCS) greater than 13, with a mean GCS of 13.9 ± 0.87 on admission. All 18 patients’ main injuries were closed femoral fractures and chest injuries. The majority of wounds were mainly caused by traffic accidents (*n* = 10), followed by falls (*n* = 5) and crushes (*n* = 3) (Tables 1 and 2). Additionally, 18 healthy volunteers, matched for age and sex, were recruited for control group.

**Table 1:** Patient’s characteristics.

Severe Trauma Patients (*n* = 18)	
Age, median (IQR), y	45 (35–54)
Sex, No. (%)	
Female	8 (44))
Male	10 (56)
Mechanism of injury, No. (%)	
Traffic accidents	10 (55.6)
Falls	5 (27.8)
Crushes	3 (16.6)
Glasgow Coma Scale score, median (IQR)^a^	14 (13–15)
Injury Severity Score^b^ Median (IQR)	20.5 (19–21)

*IQR* inter-quartile range.

^a^Glasgow Coma Scale score: an important scoring system for measuring the extent of impaired consciousness in trauma patients. Scores range is 3 (patient does not respond to any stimuli, including pain) to 15 (patient’s state of consciousness is at a normal level).

^b^Injury Severity Score: an established medical score to assess trauma severity with a score range from 0 (no injury) to 75 (maximum injury). The three body systems with the highest AIS score are used to calculate the Injury Severity Score. Each of these three scores are squared and results summed to produce the injury severity score.

**Table 2:** Patient’s characteristics in TICU.

Post trauma, hours (*n* = 18)	4 hours	8 hours	24 hours	*P*
SBP, median (IQR), mmHg	106 (96.5–113.5)	113 106.5–123.75)	112 (99.5–119.75)	ns
Heat rate, median (IQR), beats/min	116 (105.5–129)	103.5 (94.25–112)	101.5 (96.25–118.25)	*
Lactose level, median (IQR), mmol/L	2.8 (1.725–3.85)	3.85 (2.375–5.6)	1.9 (1.2–3.25)	*
**Cytokines levels**				
TNFα, mean (95% CI), pg/ml	8.87 (8.33–9.42)	10.08 (9.20-10.96)	12.48 (10.01–14.95)	**
IL6, mean (95% CI), pg/ml	37.75 (32.13–43.36)	48.63 (46.92-50.35)	59.08 (53.94–64.22)	**
IL-1β, mean (95% CI), pg/ml	12.02 (9.90–14.13)	10.40 (9.75–11.05)	33.21 (27.45–38.97)	**
IL-2, mean (95% CI), pg/ml	22.51 (17.18–27.84)	21.88 (16.38–27.39)	21.27 (16.81–25.74)	ns
IL-10, mean (95% CI), pg/ml	24.30 (17.93–30.66)	26.49 (21.22–36.00)	23.271 (15.96–30.59)	ns

*SBP* systolic blood pressure, *TICU* trauma intensity care unit, *IQR* inter-quartile range.

*P* values of SBP, heart rate and lactose were calculated by Kruskal-Wallis Test. *P* values of cytokines were calculated by ANOVA test. NS: no significance; *P < 0.05; **P < 0.01.

### Result 2. The dramatic loss of NK cells due to trauma-induced NK cell death

**Fig. 1:**
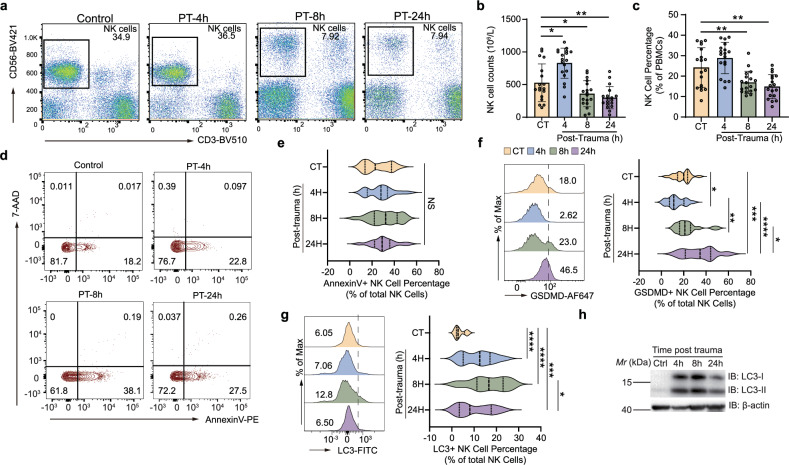
Trauma-induced NK cell depletion and apoptosis. **a** Peripheral blood mononuclear cells were isolated from healthy controls and patients at different post-trauma time points and labeled with anti-human antibodies against CD3 and CD56. Representative dot plot of CD3 and CD56 staining (gated on PBMCs of patients or healthy controls). The absolute number of NK cells (**b**) and the percentage of NK cells (**c**) were assayed in healthy controls (*n* = 13) and traumatic patients (*n* = 18) at different time points (PT-4h, PT-8h, and PT-24h) after injury. The results represent the mean ± SEM of independent experiments. ***p < 0.001 according to an unpaired, two-tail Student’s t-test. Representative flow cytometry countor plot (**d**) and violin plot (**e**) indicate the proportion of Annexin V^+^ 7-AAD^−^ NK cells. Representative flow cytometry histogram and violin plot indicate the proportion of GSDMD^+^ NK cells (**f**) and LC3^+^ NK cells (**g**) in the total NK cells of healthy individuals and severe trauma patients at different time points. The colors represent different post-trauma time points (PT-4H, PT-8H, and PT24H). Violin plots show the probability density distribution of Annexin V^+^ 7-AAD^-^ NK cells (**e**), GSDMD^+^ NK Cells (**f**) and LC3^+^ NK Cells (**g**) as a percentage of total NK cells, where the three vertical lines indicate the quartiles and medians, with *p* values (***p < 0.001, **p < 0.01, *p < 0.05) calculated by an unpaired, two-tail Student’s t-test. **h**, Immunoblot of LC3 in purified NK cells from another cohort of trauma patients (*n* = 30, PT-4h, PT-8h and PT-24h). PT-4h post-trauma 4 h, PT-8h post-trauma hours, PT-24h post-trauma 24 hours, NK natural killer, NS not statistically significant, SEM standard error of the mean.

Peripheral blood was collected from healthy volunteers or severe trauma patients at 4, 8, and 24 h post trauma, and alterations in peripheral blood mononuclear cells (PBMCs) were detected using flow cytometric analysis (FCA). Compared to healthy volunteers, both the absolute number and percentage (relative to total PBMCs) of NK cells increased at 4 h post trauma, and then decreased at 8 and 24 h in severe trauma patients (Fig. 1a–c).

Our findings were consistent with the lymphopenia observed in trauma patients [7]. Several studies suggest that apoptosis is likely to play a critical role in the clearance of overexpressed lymphocytes [5, 13]. Therefore, we conducted Annexin V and 7-AAD FCA (Fig. 1d) to evaluate the contribution of cell apoptosis to trauma-induced NK cell depletion. The percentage of AnnexinV^+^7-AAD^−^ NK cells elevated at 4 h post trauma and then gradually dropped back to normal levels at 8 h, with a slightly decreased at 24 h (Fig. 1e).

The lack of a persistent increase in the percentage of apoptotic NK cells contradicts the remarkable and rapid decrease in the number of NK cells, suggesting that in addition to cell apoptosis, other cell death paradigms are also involved in trauma-induced depletion of NK cells. To investigate the impact of non-apoptotic cell death on NK cell reduction after severe trauma, we specifically chose to observe the two most common non-apoptosis pathways: pyroptosis and autophagy, both of which have been reported to exert a pivotal effect in tumor-induced NK cell depletion [16, 17].

PBMC samples from the same batch isolated from healthy volunteers or severe trauma patients were analyzed to evaluated pyroptosis and autophagy in NK cells. GSDMD (Gasdermin D) is widely used in the characterization of cellular pyroptosis. It mediates the formation of pores in the cell membrane causing osmotic potential disruptions that lead to cell swelling and lysis [18]. The finding of flow cytometry showed that the proportion of GSDMD^+^ NK cells decreased 4 h post trauma and then increased at 8 and 24 h (Fig. 1f). LC3 (Microtubule-associated protein 1 A/1B-light chain 3) is a sensitive and effective marker for detecting autophagy in cells [19]. Flow cytometry analysis shows that the proportion of LC3^+^ NK cells peaked at 8 h post trauma with a subsequent persistent decline (Fig. 1g). In a validated independent cohort (Supplementary Table 1), we isolated NK cells from patients at various time points using flow cytometry and examined the expression of LC3 in NK cells. Our findings indicated that LC3 was significantly elevated at 4 and 8 h post injury and declined at 24 h post injury (Fig. 1h). These findings indicated that multiple cell death paradigms are involved in the process of severe trauma-induced NK cell depletion.

### Result 3. The different paradigms of NK cell death after traumatic insults

To comprehensively understand the various paradigms of NK cell death post trauma, 12 different RCD signaling pathways were simultaneously detected and analyzed by single-cell RNA sequencing (scRNA-seq) technologies. The recent breakthrough in scRNA-seq technology permits exhaustive investigation into the various paradigms of cell death. It will provide a global view of the underlying molecular mechanisms in NK cell depletion induced by severe trauma.

**Fig. 2:**
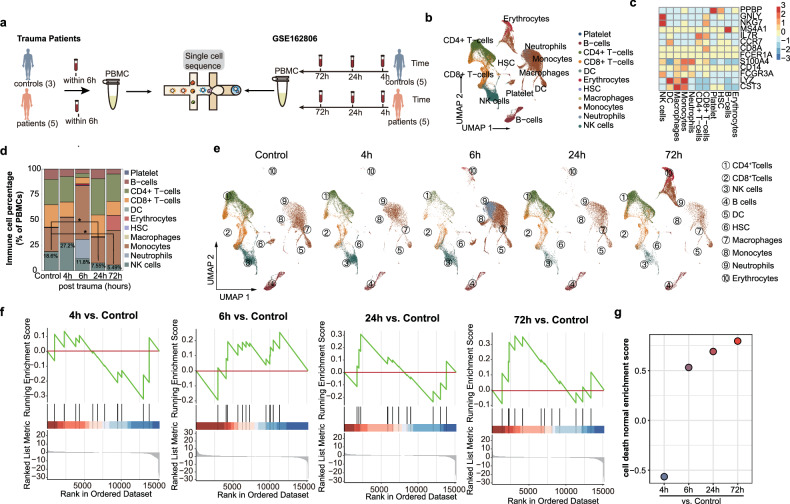
scRNA-seq identifies trauma-specific immune-cell states and gene signatures. **a** Schematic diagram detailing trauma single-cell sequencing cohort construction. **b** Eleven distinct blood immune cells were numbered, named, and displayed with a uniform manifold approximation and projection (UMAP) plot. Each point represents a single-cell colored by its cell type. **c** Canonical markers of each cell type were plotted using a heatmap. Color scale corresponds to z-scored, log-transformed mean gene-expression counts for each cell type. **d** Proportion of each immune population out of all immune cells in (**a**). Each block of the bar is colored by its cell type, and the number indicates the percentage of NK cells at each time point after injury. *p* values were calculated by comparing them with healthy controls using a two-tailed Wilcoxon rank-sum test. *p < 0.05; **e** UMAP plots for each post-trauma time point (*n* = 34423, 19195, 31752, 18380, and 19195 cells for control, post-trauma 4 h, post-trauma 6 h, post-trauma 24 h and post-trauma 72 h, respectively), colored and labeled by cell types. **f** Cell death gene set enriched in the post-trauma 4, 6, 24, and 72 h patients compared to the healthy controls. **g** Bubble plot of cell death gene set normalized enrichment score (NES) across different time points compared to healthy controls. Different groups colored each bubble. DC dendritic cell, NK natural killer, HSC hematopoietic stem cell, GO Gene ontology.

Recently, Dr. Timothy R. Billiar firstly shared severe trauma patients’ scRNA-seq data generated by 10X Genomics Chromium system at 4, 24 and 72 h post trauma [20]. Based on our aforementioned FCA findings, we consider 6 h post trauma to be a critical time point for the initiation of multiple cell death pathways in NK cell. Therefore, we matched five severe trauma patients based on the ISS values, GSC values, age, sex and injury of patients in Dr. Timothy’s clinical cohort (Table 3). After isolating their PBMCs from peripheral blood collected at 6 h post trauma, scRNA-seq of these PBMCs was immediately generated using droplet-based scRNA-seq method on 10X Genomics Chromium single-cell platform (Fig. 2a).

**Table 3:** Basic characteristics of severe trauma patients for scRNA-seq.

Data source	Patient ID	ISS	Age	Sex	Injury	Admission chemistry		
						SBP	GCS	Lactate
GSE162806^19^	MM3001	22	21	Male	MVC	71	15	4.8
	MM3008	19	26	Male	Penetrating	70	15	6.9
	MM3009	13	71	Male	Fall	88	15	5.8
	MM3016	18	32	Male	MVC	127	15	2.4
	MM3020	27	78	Male	Fall	83	8	NA
Matched patients for scRNA-seq	Patient01	21	25	Male	MVC	84	15	3.4
	Patient02	19	30	Male	Fall	89	15	1.2
	Patient03	18	60	Male	Fall	98	15	3.8
	Patient04	18	34	Male	MVC	114	14	4.2
	Patient05	25	55	Male	Fall	82	13	6.9

*ISS* Injury severity score, *MVC* motor vehicle collision, SBP Systolic blood pressure, *GCS* Glasgow Coma Scale score, *NA*, not available.

To remove batch effects, we used the anchor-based fast integration using reciprocal Principal component analysis (RPCA) integration method to integrate these two based droplet-based scRNA-seq data [21]. The integrated result showed a consistent distribution of scRNA-seq data across batches, indicating that the batch effect has been eliminated (Supplementary Fig. 1a). Following quality control and batch effect correction, transcriptional profiles were collected from 133,029 cells, categorized as control (*n* = 34,423), 4 h post trauma (PT-4h, *n* = 19,195), 6 h post trauma (PT-6h, *n* = 31,752), 24 h post trauma (PT-24h, *n* = 18,380), and 72 h post trauma (PT-72h, n = 29,279) (Fig. 2b). A total of twelve distinct major cell lineages of trauma-induced PBMCs were identified, which comprised NK cells (GNLY^+^NKG7^+^), dendritic cells (DC, FCER1A^+^CST3^+^), macrophage (CST3^+^), monocytes (CD14^+^LYZ^+^), neutrophils (S100A4^+^), CD4^+^ T cells (IL7R^+^CCR7^+^S100A4^+^), CD8^+^ T cells (CD8A^+^), and B cells (MS4A1^+^) (Fig. 2c and Supplementary Fig. 1b).

After defining these clusters using data from all subjects, the differences in abundances of these cells across different post-trauma time points were analyzed using cell percentage analysis (Fig. 2d). In severe trauma patients, NK cells underwent the most pronounced variations among all immune cells (Fig. 2d, e). NK cells were found to be present at slightly higher levels in PT-4h patients compared to healthy controls. However, they significantly declined in PT-24h and PT-72h patients (Fig. 2d, e). The trend of NK cells detected by scRNA-seq was consistent with flow cytometry analysis. Gene Set Enrichment Analysis (GSEA) indicated that the cell death pathway of NK cells was enriched at 6, 24, and 72 h after injury (Fig. 2f). The cell death pathway was downregulated at 4 h and upregulated at 6 h after trauma, which is corresponding to the alteration of the NK cell numbers increased within PT-4h and decreased at PT-6h (Fig. 2g).

To further clarify the signaling pathways of cell death, we collected gene expression signature profiles of twelve cell death pathways from multiple databases including Gene Ontology (GO), Kyoto Encyclopedia of Genes and Genomes (KEGG), Reactome, and Wiki Pathways (Supplementary Table 2). According to the recommendations of the Cell Death Nomenclature Committee [22], these twelve paradigms of cell death included intrinsic apoptosis, extrinsic apoptosis, mitochondrial permeability transition-driven necrosis, necroptosis, ferroptosis, pyroptosis, parthanatos, entotic cell death, neutrophil extracellular traps cell death, lysosome-dependent cell death, autophagy-dependent cell death, and immunogenic cell death.

**Fig. 3:**
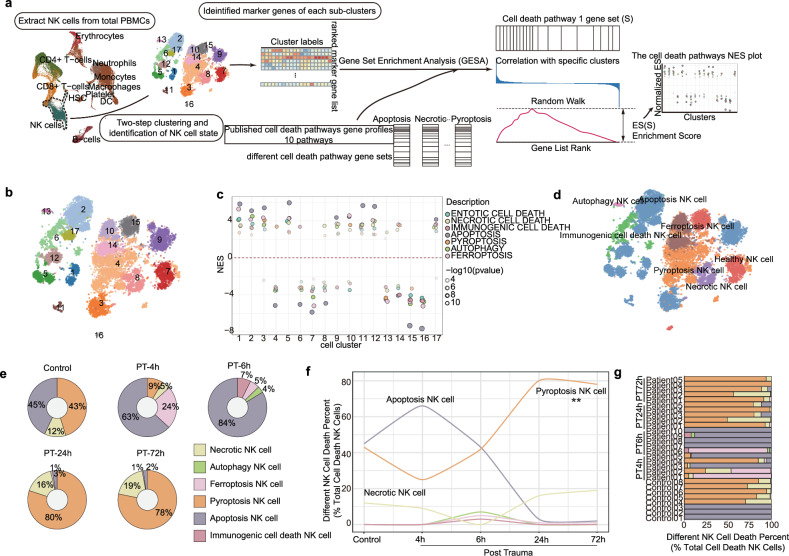
scRNA-seq defined NK cell state in severe trauma patients. **a** Cell death analysis pipeline for NK cells. NK cell states were identified via two-step clustering, and cluster difference thereof compared to find death-specific states of NK cells. Further cell death signatures were derived from these states using gene set enrichment analysis (GSEA). **b** T-distribution stochastic neighbor embedding (t-SNE) plots for each cluster. Each point was colored by the cluster to which it belonged. **c** Ten cell death pathways in the differentially accessible genes of each cluster. Sizes of circles are proportional to the normalized enrichment score (NES). Color represents the cell death pathways, and alpha represents the log10 *p* value. *p* value was calculated by empirical phenotype-based permutation test procedure according to the Kolmogorov-Smirnov-like statistic. **d** t-SNE plots for each cell state (Autophagy NK cell, apoptosis NK cell, ferroptosis NK cell, immunogenic cell death NK cell, pyroptosis NK cell, Necrotic NK cell, and healthy NK cell), and each point was colored by its cell state. **e** Pie charts displaying the fraction of cells in six cell death states in healthy controls and different post-trauma time point trauma patients. **f** The line graphs illustrate variations in the percentage of NK cells with different death paradigms at various post-trauma time points. Colors distinguish between the different paradigms of death while *p* values were computed utilizing one-way ANOVA (*p < 0.05, **p < 0.01, ***p < 0.001). **g** Stacked bar plot showing the fraction of each patient in each of the 6 cell death states. Different colored blocks represent the diverse methods of NK cell death, and the colors convey the same meaning as in the illustration displayed in (**e**).

After re-clustering the NK cells using a two-step procedure involving vector quantization with k-means as the first step and graph-based clustering as the second step, NK cells were categorized into 17 distinct clusters with their own unique marker genes (Fig. 3a, b). Normalized enrichment scores (NES) for the 12 cell death genes were calculated by GSEA among these DEGs (differential expressed genes) in each cluster individually. Pathways with the highest NES were chosen as the cell death paradigm for the target clusters (Fig. 3c). The apoptosis cell death pathway was enriched in clusters 1, 2, 3, 5, 9, 11, 12, and 17. The pyroptosis cell death pathway was enriched in cluster 4. The autophagy cell death pathway was enriched in cluster 13. The ferroptosis cell death pathway was enriched in clusters 6, 10, and 14. The necrotic cell death pathway was enriched in cluster 8. No cell death pathway was enriched in clusters 7, 15, and 16, and we identified these clusters as healthy NK cells (Fig. 3c). The distribution of these NK cell states was intuitively shown in the t-SNE plot (Fig. 3d).

After defining the states of NK cells, we analyzed the differences in the abundance of these cell states across various groups (Fig. 3e). At the onset of trauma (4 and 6 h), apoptosis was the main pathway for cell death and may contribute to NK cell depletion, with 63% at 4 h and 84% at 6 h. Consistent with our flow cytometric results that with the progression of trauma, pyroptosis has replaced apoptosis as the main pathway leading to NK cell depletion, with 80% at 24 h and 78% at 72 h (Fig. 3f, g). Intriguingly, necrotic NK cell death increased gradually at 24 h post injury. Signaling pathways for immunogenic cell death, ferroptosis, and autophagy are upregulated at 6 h post trauma. These findings suggest that NK cells undergo several cell death pathways after severe traumatic injury, dominated by apoptosis and pyroptosis, accompanied by a small amount of necrotic cell death, immunogenic cell death, ferroptosis and autophagy.

### Result 4. Hypoxia is a pivotal factor in the diversification of NK cell death patterns

**Fig. 4:**
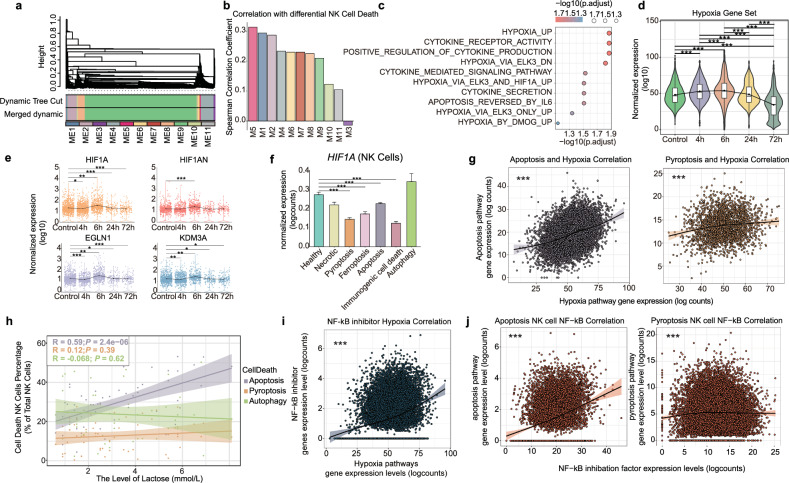
Hypoxia is the determiner of intrinsic NK cell death alteration. **a** Weighted gene co-expression analysis hierarchical clustering dendrogram of the genes that passed quality control filtering for trauma patients. Gene dissimilarity was calculated using a topological overlap measure. The colors corresponding to network assignments are given in the bar below the dendrogram. **b** The bar plot shows the correlation between the pseudo time of NK cells and traumatic network eigengene values using Spearman methods. The color of each bar represents the module color. **c** Pathway enrichment analysis showing the top ten enriched pathways for module 5. Sizes of circles are proportional to the number of genes hits in a set, whereas color represents the enrichment score of each gene set. **d** Violin plot demonstrates the expression levels of the hypoxia signaling pathway within NK cells at different post-trauma time points. The violin plot shows the probability density distribution of the hypoxia pathway expression level, while the box plot within the violin shows the median and quartiles, and the whisker of box plot shows the 95% confidence interval. **e** The dot plots show normalized gene expression for each HIF-1α-related gene in each post-trauma time point (HIF1A, HIF1AN, EGLN1, and KDM3A). Black dots represent the mean values of target gene expression. **f** The bar plot shows normalized gene expression of HIF-1α in each cell death state. *p* values were calculated using a two-tailed Wilcoxon rank-sum test. *p < 0.05, **p < 0.01, ***p < 0.001. **g** The correlation between hypoxia pathway genes and apoptosis genes (left panel) and pyroptosis genes (right panel). The lines represent loess regression fit curves, and the shading of the lines demonstrates the 95% confidence intervals. *p* value was calculated by one-way ANOVA method. ***p < 0.001, **p < 0.01, and *p < 0.05. **h** Linear regression analysis shows the correlation between lactate levels and the proportion of NK cells with different paradigms of death; different colors represent NK cells with different paradigms of death. The shading represents 95% confidence intervals, *R* value represents linear correlation coefficient, and *p* value was obtained by one-way ANOVA. ***p < 0.001, **p < 0.01, and *p < 0.05. **i** The correlation between hy*p*oxia pathway genes and NF-kB inhibitor genes (NFKBIA and NFKBIB). and *p* value was obtained by one-way ANOVA. ***p < 0.001, **p < 0.01, and *p < 0.05. **j** The correlation between NF-kB pathway genes and apoptosis (left panel) and pyroptosis (right panel). The fitted curves were obtained using loess regression. Shaded areas show 95% confidence intervals for the fitted curves. and *p* value was obtained by one-way ANOVA. ***p < 0.001, **p < 0.01, and *p < 0.05.

To identify potential factors influencing the diversity of NK cell death, the weighted gene co-expression network analysis (WGCNA) was performed on scRNA-seq of PBMCs from severe trauma patients. Based on an average clustering algorithm, a total of 11 modules consisting of the similar expressed genes under different conditions were obtained (Fig. 4a). The eigengenes of the module were screened using principal component analysis to represent the gene module. Eigengenes are considered to be the weighted average expression profile of all genes in a module. The expression of the eigengenes represents the expression level of this module [23]. Spearman correlation analysis between the expression of module eigengenes and different cell death patterns revealed that eigengene of module 5 (M5) had the strongest correlation among eigengenes of other modules (Fig. 4b). To explore the biological properties of the M5 genes, an enrichment analysis of cell signaling pathways was performed. Our findings revealed that the hypoxia signaling pathway has the most significant impact on the diversity of NK cell death (Fig. 4c).

To investigate the degree of hypoxia in patients at different time points post-trauma, we explored the expression levels of hypoxia-related genes in NK cells of severe trauma patients. Hypoxia signaling pathways cumulate progressively within 6 h after injury in severe trauma patients. While at 24 h and 72 h post trauma, the expression of hypoxia signaling pathways in vivo declines gradually (Fig. 4d). HIF-1A (hypoxia-inducible factor 1-alpha) is recognized as a key transcriptional regulator of the cellular and developmental response to hypoxia [24, 25]. The expression level of its encoding gene (*HIF1A*) peaks at 6 h post trauma and declines thereafter (Fig. 4e). In validation cohort (Supplementary Table 1), we corroborated these findings through qRT-PCR, which indicated that the mRNA level of HIF-1A reached its peak at 8 h post injury, subsequently declining at 24 h post injury (Supplementary Fig. 2a). Similarly, the expression levels of genes encoding HIF-1 regulators [26] (*HIF1AN*, *EGLN1*) and its effector [26] (*KDM3A*) were also elevated at 6 h post trauma, followed by a sustained decline (Fig. 4e). Lactate, as an important indicator of the hypoxic levels in vivo [27], also significantly increased at 8 h post trauma and declined at 24 h in the cohort of 18 severe trauma patients (Table 2). These data demonstrated that the degree of hypoxia in severe trauma patients rises in the hyperacute phase after injury, with a moderate improvement in hypoxic levels later.

Hypoxia is associated with numerous paradigms of cell death [28–32]. We extracted NK cells with different paradigms of cell death to explore the relation between different cell death signaling and hypoxia, respectively. The expression of *HIF1A* was significantly upregulated in autophagic NK cells and inhibited in NK cells with pyroptosis and immunogenic cell death (Fig. 4f). Loess regression analysis suggested that the links between distinct cell death signaling pathways and hypoxia-related genes were different (Fig. 4g and Supplement Fig. 2b). Specifically, gene expression levels of apoptosis and ferroptosis were elevated with enhancement of the hypoxia signaling pathway in NK cells. In contrast, gene expression of other cell death pathways including pyroptosis, necrotic, immunogenic cell death and autophagy entered a distinct plateau with the enhancement of the hypoxia signaling pathway in NK cells (Fig. 4g and Supplementary Fig. 2b). Consistently, lactate levels were significantly and positively correlated with the proportion of apoptotic NK cells, whereas there was no significant correlation with the proportion of pyroptotic or autophagic NK cells (Fig. 4h).

In order to further explore the pivotal molecules involved in the differential response of different cell death’s NK cells to hypoxia, correlation analysis was performed between all death molecules and hypoxia-related genes. Our finding showed that the NF-kB (nuclear factor kappa-B) related genes were most significantly correlated with hypoxia (Supplementary Fig. 3). NF-kB is a shared transcription factor that simultaneously regulates apoptosis and pyroptosis [33]. Further correlation analysis showed that genes encoding inhibitory molecules of NF-kB (NFKBIA, NFKBIB) were significantly positively correlated with hypoxia-related genes (Fig. 4i and Supplementary Fig. 4). Similar to the relationship between hypoxia and cell death, enhancement of the NF-kB inhibitory pathway was accompanied by an increase in apoptotic signaling, whereas pyroptotic signaling remained stagnant (Fig. 4j). Collectively, hypoxia plays an important role in the diversity of NK cell death, and the different death paradigms in NK cells exhibit distinct relationships to hypoxia.

### Result 5. Diversity of cytokine activation profiles in NK cells with different death paradigms

**Fig. 5:**
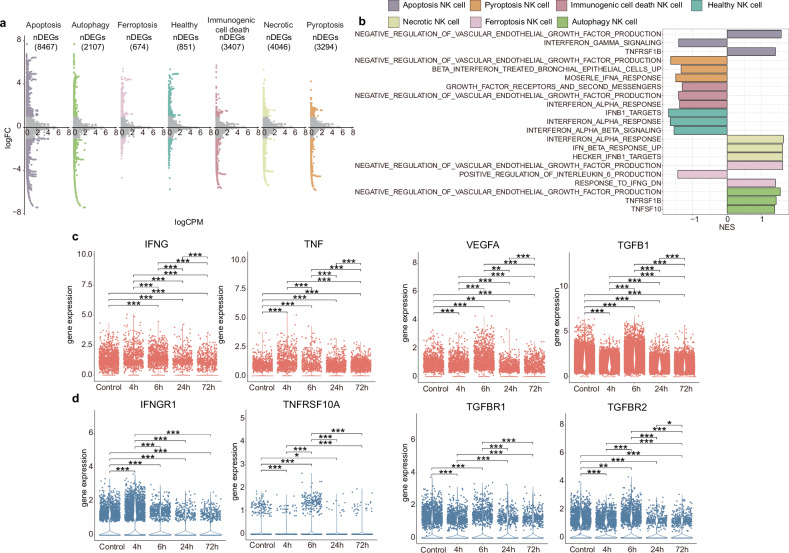
The effect of cytokines in various NK cell death. **a** Ratio-intensity plots demonstrate the presence of differential genes in NK cells with distinct death mechanisms (genes having logFC > 1 or < −1 were distinguished as the differential genes). Color coding is used to represent differential genes of various death patterns, while the number of differential genes is displayed within parentheses. **b** Bar plot showing normalized enrichment scores of cytokine pathways for differential expressed genes. Each bar was colored by different cell death states. **c** Scatter plots of different cytokines genes detected from PBMCs across different post-trauma time points. **d** Scatter plots of different cytokine receptors detected from NK cells across different post-trauma time points. *p* value was calculated using an unpaired, two-tail Student’s t-test. *p < 0.05, **p < 0.01, ***p < 0.001. DEGs differentially expressed genes.

In light of the results of the M5’s enrichment analysis (Fig. 4c), it is reasonable to hypothesize that the cytokines expression profiles of NK cells with different paradigms of death are diverse. To clarify the differences in cytokine expression profiles of NK cells with different paradigms of death, we employed a pairwise comparison strategy to obtain the differentially expressed genes (DEGs) for downstream analyses (Fig. 5a). Among them, 8467 DEGs were identified in apoptotic NK cells, 2107 DEGs in autophagic NK cells, 674 DEGs in ferroptosis NK cells, 851 DEGs in healthy NK cells, 3407 DEGs in immunogenic cell death NK cells, 4046 DEGs in necrotic NK cells, and 3294 DEGs in pyroptosis NK cells (Fig. 5a). The GSEA of cytokine signaling pathway was performed on aforementioned DEGs, and the result suggests a significant difference in the activation of cell signaling pathways in NK cells of different death paradigms (Fig. 5b).

Specifically, within apoptotic NK cells, interferon and especially interferon gamma signaling was suppressed. In contrast, the signaling pathway inhibiting vascular endothelial growth factor (VEGF) production and the TNFRSF1B signaling pathway were significantly up-regulated. In pyroptotic cells, the declining interferon (IFN) signaling pathway, especially IFNα response, is specifically conspicuous. Growth factors, IFNA and VEGF negative regulation signal pathways were dampened in immunogenic NK cell death. In addition, necrotic NK cells are accompanied by elevated levels of IFNα and IFNγ. IFNG and VEGF negatively regulation signaling pathways were activated in ferroptosis NK cells, while IL6 signaling pathway was suppressed. The VEGF inhibitory regulatory signaling pathway and TNF signaling pathway are activated in autophagic NK cells. Moreover, in terms of healthy NK cells, the expression of IFN-related signaling pathways were all downregulated, including IFNα and IFNβ signal pathways (Fig. 5b).

In order to investigate the changes in cytokine secretion and cell surface receptors of NK cells at different post-injury time points, we extracted the cytokine expression profiles of NK cells. mRNA analysis demonstrated that the expression of NK cell-secreted IFNγ, TNF, VEGFA, and TGFB1 was elevated in the ultra-early post-trauma period (PT-4h and PT-6h), and its expression was gradually decreased at 24 h and 72 h post trauma (Fig. 5c). In validation cohort of severe trauma patients (Table 3), NK cells from patients at different time points post-trauma were sorted and verified for mRNA expression of cytokines based on flow cytometric cell sorting. The results of qRT-PCR indicated that cytokine secretion was elevated in NK cells during the ultra-early post-injury period, while there was no discernible difference in cytokine secretion at 24 h post injury compared to that of the control group (Supplementary Fig. 5a–d). The results of mRNA expression analysis of NK cell surface receptors indicated that the expression of their corresponding cytokine receptors (IFNGR, TNFRSF10A, TGFBR1, and TGFBR2) increased in the ultra-early post-injury period. However, the expression level declined at 24 h post injury (Fig. 5d). These results were also validated in the cohort using qRT-PCR, which indicated that extracted mRNA for the IFNγ receptor (IFNGR1), TNF receptor (TNFRSF10A), and TGFBR1 on the surface of NK cells exhibited elevated expression at 8 h post trauma. In contrast, no significant difference was observed in the mRNA expression of TGFBR2 (Supplementary Fig. 5e–h). Together, these data demonstrated a different profile of cytokine signaling pathways activated in NK cells of different death paradigms. Moreover, the gene expression levels of these critical cytokines and their receptors vary with the progression of severe trauma.

### Result 6. Intercellular communication networks between NK cells with different death paradigms and various immune cells

**Fig. 6:**
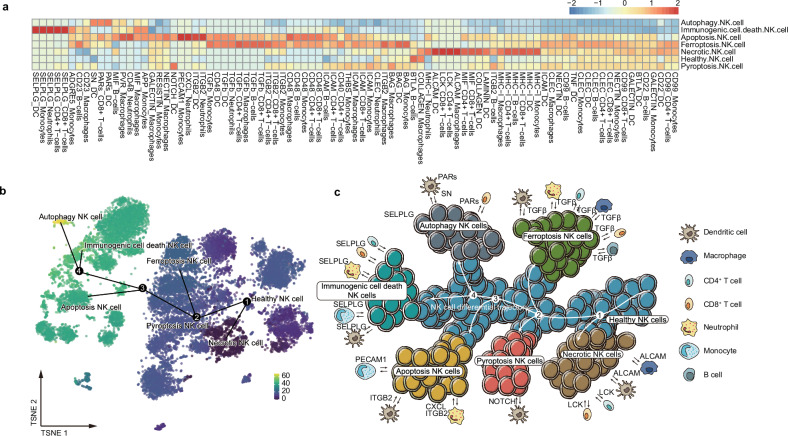
External NK cell environment: cell trajectory and cell communication. **a** Heat map shows the weight of different cell-cell communication pathways from different cell types. Color represents the weights of NK cells with different paradigms of death communicating with other immune cells in signaling pathways. **b** TSNE plot showing differential trajectory inferred by tools for single-cell analysis (TSCAN) algorithm, where each point is a cell and is colored according to its pseudotime value. The minimum spanning tree (MST) obtained using TSCAN with mutual nearest neighbor (MNN) distances is overlaid on top. The numbers indicated decision points. **c** Schematic diagram showing the NK cell differentiation pathway and the interaction of NK cells with other immune cells in different ways of death.

Considering the diversity function in NK cells with different death paradigms, it is important to explore the effects of these functionally diverse NK cells on the entire immune system of severely injured patients. CellChat [34], a tool capable of inferring and analyzing intercellular communication networks from single-cell data, was used to depict the communication networks of NK cells in diverse death states with external cells. Following the calculation of the weights of communication between different cell death NK cells and other immune cells by CellChat, it was revealed that apoptotic NK cells had the more powerful interaction with neutrophils, dendritic cells and monocytes than other immune cells (Fig. 6a). Specifically, the chemokine (C-X-C motif) ligand (CXCL) signaling pathway (mediated by neutrophils), the integrin subunit beta 2 (ITGB2) signaling pathway (mediated by neutrophils and dendritic cells) and the platelet and endothelial cell adhesion molecule 1 (PECAM) signaling pathway (by monocytes) were given top weight among the signals that intercommunicate with apoptotic NK cells and other cells (Supplementary Fig. 6a, b). Pyroptotic NK cells have the weakest network connectivity with other immune cells and communicate mainly with dendritic cells through the NOTCH signaling pathway (Supplementary Fig. 6c). In regards to necrotic NK cells, they are primarily affected by two signaling pathways, activated leukocyte cell adhesion molecule (ALCAM) and tyrosine-protein kinase (LCK), which are coupled to various immune cells included dendritic cells and T cells (Supplementary Fig. 6d, e). Autophagy NK cells communicate with dendritic cells and CD8^+^ T cells via protease activated receptors (PARs) and secretoneurin (SN) pathways (Supplementary Fig. 6f. g). Immunogenic cell death NK cells communicate with dendritic cell, T cell, monocyte, and neutrophils though selectin p and its ligand (SEPLG) signaling pathway (Supplementary Fig. 6h). Ferroptotic NK cells communicate with various immune cells (dendritic cell, macrophage, T cell, neutrophil, monocyte and B cell) via transforming growth factor beta (TGFβ) signaling pathway (Supplementary Fig. 6i).

NK cells in distinct states of cell death are suffering from both external cellular effects as well as undergoing their internal natural differentiation procedures. Trajectory inferred by tools for single-cell analysis (TSCAN) algorithm [35] was performed on the various NK cell death and it showed that NK cell trajectory constitutes four decision points and seven states. With the differentiation of NK cells, the various cell death NK cells have taken up different branches of the differentiation trajectory. Differentiation of NK cells initiate from healthy NK cells, which diverge at the first differential decision point into NK cells that are susceptible to necrotic cell death. NK cells at the second decision point of differentiation are expected to differentiate into ferroptotic NK cells and pyroptotic NK cells. NK cells at the third differentiation decision point will differentiate into apoptotic NK cells. At the fourth differential decision point, NK cells are supposed to differentiate into immunogenic cell death’s NK cells and autophagic NK cells (Fig. 6b). Taken together, our data provided a panoramic view of communication and differentiation of NK cells with different paradigms of death in the immune environment of severe trauma patients (Fig. 6c).

## Discussion

Following improvements in medical care, the amount of trauma patients who die from early uncontrollable hemorrhage has gradually declined [1]. The subsequent delayed death from fatal infections due to immune suppression has become the greatest concern in the management of severe trauma patients at present [6, 36]. Over the past few decades, considerable efforts have been devoted to exploring severe trauma-induced immunosuppression [37, 38]. However, the contradictory findings have always been a major barrier to achieve widely reproducible clinical benefits.

In the present study, the findings indicated that severe trauma patients underwent post-injury immunosuppression, primarily in the form of lymphopenia, with the most significant reduction in NK cells. NK cells served as the first line of immune defense and are essential for immunosurveillance during tissue trauma and infection [11, 39]. After trauma, activated NK cells via DAMPs enhance in vivo resistance to pathogenic aggression [11]. Persistent declines in NK cells have been demonstrated to be associated with a poor prognosis and a high risk of mortality in severe trauma patients [9, 40–42]. Nevertheless, the underlying mechanisms causing NK cell reduction are poorly understood.

The reduction in NK cells in PBMC is frequently caused by redistribution or cell death. Recent studies have shown no evidence of NK cell migration to potential sites such as wounded tissue, lung, spleen and lymph nodes after trauma [12, 13]. Indeed, it is now well established that cell death is the major cause for the dramatic loss of circulating NK cells after severe trauma. In the past, Lymphopenia induced by severe trauma is frequently attributed to cells apoptosis. However, intervening in apoptosis of NK cells does not completely reverse the continued depletion of NK cells during severe trauma, which is inconsistent with conventional thought in NK cell reduction caused by apoptosis [14].

In the present study, our findings confirmed that cell death pathway was significantly activated in NK cells following injury. More importantly, we found that apoptosis does not perfectly explain the trauma-induced NK cell depletion, and that multiple cell death pathways are involved in this process. Specifically, NK cell death is a diverse paradigm dominated by pyroptosis and apoptosis, with coexistence of other ways of cell death including necrotic cell death, ferroptosis, autophagy and immunogenic cell death. Notably, in the hyperacute phase of severe trauma, the paradigm of death in NK cells is dominated by apoptosis. During the progression of trauma, pyroptosis gradually became a major cell death pathway contributing to the depletion of NK cells. NK cells undergoing pyroptosis will suffer cellular swelling and plasma membrane rupture, promoting the release of pro-inflammatory cytokines IL-1β, IL-18, and cellular contents into the extracellular space, thereby activating the inflammatory response [43]. Consistently, pro-inflammatory cytokines increased gradually within 24 h post injury, with the most dramatic increase in IL-1β. TNFα and IL6 also had a modest increase (Table 2). Current research suggests synergistic increases in IL-1β and IL6, TNFα after severe trauma will lead to a worse outcome for the patients [44]. The release of these pro-inflammatory cytokines may be related to the diversity of cell death paradigms. The diversity of NK cell death on the one hand increases the phenomenon of immunosuppression and on the other hand promotes the release of pro-inflammatory cytokines in severe trauma patients. This may help to explain the paradoxical coexistence of pro-inflammatory response and immunosuppression in severe trauma patients. Meanwhile, the diversity of NK cell death paradigms prompts that blocking a single-cell death pathway will probably not help to prevent the depletion of NK cells. Identification of the potential pathway to simultaneously interfere with multiple cell death would be tremendously effective in alleviating immunologic derangement caused by the decline in NK cell populations.

In order to clarify this potential pathway, in the present study, our findings suggested that hypoxia accounted for the pivotal factor in the diversification of NK cell death. After the occurrence of severe trauma, there is an oxygen debt due to hemorrhagic shock, coagulopathy or endothelial damage [45–47]. This is consistent with the fact that the hypoxia signaling pathway is activated and its expression is up-regulated in NK cells at 4 and 6 h post trauma, when apoptosis dominates NK cell death. Subsequently, sustained resuscitation interventions alleviated hypoxia in trauma patients. The expression levels of hypoxia-related genes were downregulated in NK cells at 24 and 72 h post trauma, when NK cell death was predominantly pyroptosis. Recent studies have suggested that hypoxia induces both pyroptosis and apoptosis [18, 28, 48]. However, the divergent point of apoptosis and pyroptosis induced by hypoxia were not discussed by these studies. Our research suggested that the different responses of NK cells to hypoxia result in diverse cell fate, and then the underlying mechanism needs to be further explored in the future.

In addition to suggesting the relevance of hypoxia to NK cell death, our findings also suggested a significant correlation between cytokine signaling pathways and the paradigm of NK cell death. Through signaling pathway enrichment analysis, it has been discovered that IFN signaling was suppressed in both apoptotic and pyroptotic NK cells. However, in contrary to pyroptotic NK cells, the TNF signaling pathway was highly enriched in apoptotic NK cells. High activation of the TNF signaling pathway leads to NK cells to entering aerobic glycolysis which impairs their proliferation and antiviral resistance [49]. The interferon has specific effects that promote NK cell expansion and may protect them from cell death [50]. The reduced expression of the interferon pathway in post-trauma NK cells indicates a lapse in protection and a raised risk of cell death.

The interaction of NK cells with various immune cells is pivotal in comprehensively priming the immune system to enhance resistance to pathogenic invasion. Our study provides a panoramic view of how NK cells with different paradigms of death are interacted with diverse immune cells. Among the series of signals by which NK cells interact with other immune cells, the most prominent communication signal is cell adhesion signaling, including ALCAM [51], ITGB2 [52], PECAM1 [53] and SELPLG [54]. Intriguingly, ferroptotic NK cells were strongly correlated with the immunosuppressive signal TGFβ. TGFβ [55], as an enforcer of immune homeostasis and tolerance, induces immune suppression within the tumor microenvironment. The high secretion of TGFβ may be associated with the inhibition of NK cells’ function [56]. Apoptotic NK cells highly express the chemotaxis signal CXCL, a chemokine which recruits and migrates different categories of immune cells [57]. NOTCH signal has been demonstrated to play an important role in promoting pyroptosis [58, 59]. It is a pivotal signaling pathway that regulates cell fate [60, 61]. This signal mediates the association of pyroptotic NK cells with dendritic cells. PARs signaling is a critical alarm signal during microbial invasion which promotes the release of a range of cytokines and chemokines by sensing proteinases [62]. This signal mediates the connection of autophagic NK cells to dendritic cells and CD8^+^ T cells. In total, this panoramic view comprehensively and intuitively exhibits the intercellular communication networks between NK cells with different death paradigms and various immune cells after severe trauma.

It is notable that during the communication of NK cells with other immune cells in different pathways of cell death, there is not only the activation of inflammation, but also the expression of immune suppressive signals. The suppressive signaling pathways include TGFβ, SELPG, and PECAM1. TGFβ is a pleiotropic cytokine that has been linked to immunosuppression and inflammatory immune response [63]. TGFβ inhibits the cytotoxicity of NK cells and cytotoxic T lymphocytes (CTL) [64, 65], while promoting the proliferation of immunosuppressive cells, such as regulatory T lymphocytes (Treg) [66–68]. PECAM1 has been demonstrated to enhance TGFβ mediated T cell suppression [69]. The enhancement of TGFβ by PECAM1 serves to reinforce the immunosuppressive function of TGFβ, ultimately resulting in an immunosuppressive state in severely traumatized patients. SELPG is also a crucial regulator of immune checkpoints that stimulates the exhaustion of immune cells [70]. In addition to the decline in the number of NK cells, the combined effects of released suppressive cytokines undoubtedly exacerbate severe post-traumatic immunosuppression.

Taken together, this study confirmed the decline of NK cells after severe trauma were mainly caused by NK cell death with different paradigms. NK cells of severe trauma patients underwent several cell death pathways predominated by apoptosis and pyroptosis pathways, with coexistence of other cell death paradigms including autophagy, ferroptosis, necrotic cell death and immunogenic cell death pathway. NK cells with distinct death paradigms show diversity in their abilities of cytokine secretion and interactions with other immune cells. Such differences may further exacerbate the development of immune disorders in severe trauma patients. Furthermore, our study revealed that hypoxia was strongly associated with this diverse paradigm of NK cell death, which suggested oxygen levels maybe a key regulator of NK cells fate. It is possible to intervene in NK cell death by regulating blood oxygen levels, ultimately alleviating immune disorders in trauma patients. Further investigation into the specific mechanisms that link hypoxia to the different paradigms of NK cell death need to be conducted in the future.

## Methods

### Patient information

From January 2021 until February 2022, eligible patients with severe trauma were admitted to the TICU of Tongji Hospital who fulfilled the severe trauma criteria. The diagnostic criteria for severe trauma were based on published guidelines [71, 72]. Patients with ISS greater than 16 were defined as severe trauma patients. These Patients’ main injuries were closed femoral fractures and chest injuries. Inclusion criteria also included: age between 18 and 60 years; and length of admission within 3 h of time of injury. The exclusion criteria were as follows: active malignancy; infected with HIV; received immunosuppressive therapy with 24 h of admission. In addition, none of these patients received corticosteroids or other immune regulatory agents before enrollment. Control subjects were selected to ensure age and sex comparability in the healthy population without significant acute or chronic illness. In accordance with the aforementioned conditions, a further 30 patients at different time points post-trauma were recruited for the validation cohort. All patients received standard treatments according to published guidelines [71, 72]. The procedures that involved human participants received review and approval by the ethics committee at Tongji Hospital and Tongji Medical College. Patients’ legally authorized representatives or patients themselves provided written informed consent.

### Sample collection and isolation of peripheral blood mononuclear cells

Peripheral venous blood samples were obtained 4, 8, and 24 h post trauma and stored under appropriate conditions. Peripheral blood mononuclear cells (PBMCs) were separated using the Ficoll-PaqueTM Plus medium (GE Healthcare, Chicago, IL, USA) through density gradient centrifugation, as previously described [73]. After centrifugation, the PBMC layer was collected, and two washes were performed using phosphate buffer solution (PBS) at room temperature.

### Droplet-based single-cell sequencing

Our scRNA-sequencing datasets can be downloaded from the GEO database (GSE197522). For scRNA-seq, we used the Chromium single-cell platform (10X Genomics) in tandem with cell hashing. Each channel held around 10,000 cells, and we retrieved 5,000 target cells. The target cells were lysed, and the resulting RNA was barcoded using HTO barcodes via reverse transcription in individual single-cell gel beads within the emulsion. Complementary DNA (cDNA) was generated and amplified following the manufacturer’s protocol, with supplementary steps taken to amplify HTO barcodes. Quality was evaluated using an Agilent 4200 following the manufacturer’s instructions. Subsequently, cDNA libraries were sequenced on a Novaseq6000 sequencer (Illumina) at a depth of 20,000 reads per cell.

### Single-cell RNA-seq data processing

The raw data were aligned to the GRCh38 reference genome using the Cell Ranger v7.0.1 (10X Genomics, Pleasanton, CA) pipeline, which generated the unique molecular identifiers (UMIs) count matrices. The resulting filtered gene expression matrices were subsequently analyzed in R software (v4.0.1) with Seurat packages [21, 74–77] (v4.1.1). Cells of insufficient quality were excluded if they met the specified criteria. The cells must adhere to the following specifications: (1) it should comprise between 200 and 2500 gene features that are unique; (2) between 800 and 10,000 gene counts; and (3) it should contain less than 5% UMIs derived from the mitochondrial genome. The filtered matrix was normalized using global-scaling normalization through the “LogNormalize” function in “NormalizeData”. Additionally, the transcriptome RNA sequencing data was reduced in dimensionality by identifying 2000 highly variable features with the “FindVariableFeatures” function. To conduct comparative analysis of scRNA-seq across experimental conditions, the scRNA-seq integration procedure commenced with the identification of anchors between each cell pair dataset through the utilization of the “FindIntegrationAnchors” function. Following the generation of the anchors, integration was achieved by employing them to create a comparable Seurat object (labeled “integrated”) that contained the gene count matrix for each cell pair dataset. A principal component analysis (PCA) was conducted using the “RunPCA” function on data that had been linearly scaled using the “ScaleData” function. The elbow plot was employed to ascertain the optimal number of effective principal components (PCs) for discerning differences, and the top 10 PCs were selected for subsequent downstream analyses. The top 10 significant principal components were used to cluster cells, employing the FindNeighbors and FindClusters functions. The RunTSNE function was employed with the default settings to conduct t-stochastic neighbor embedding (t-SNE) for non-linear dimensional reduction.

### Cell type annotation and cluster marker identification

After conducting non-linear dimensional reduction and projecting cells into a two-dimensional space using t-SNE, they were clustered based on similarities in their gene expression profiles. To identify marker genes for each cluster, we utilized the “FindAllMarker” function with default parameters. For cluster annotation, we relied on the canonical markers of specific cell types and eliminated clusters expressing two or more canonical cell-type markers.

### Differential gene expression analysis

Following aggregated individual cell replicates to generate a pseudo-bulk RNA gene matrix, Subsequently, differential expression analysis was conducted on bulk RNA gene sets and pseudo-bulk RNA gene sets utilizing the “DESeq” function within the “DESeq2” package [78] (v1.32.1) by following the standard workflow. The Result function was used to extract log2 fold change (log2FC), *p* values, and adjusted p values. Gene expression differences were deemed important and significant if the adjusted p value was less than 0.05 and the absolute value of log2 fold change value was greater than 1. Volcano plots were generated using the ggplot2 package (v3.3.6).

### Functional gene enrichment analysis

Gene set enrichment analysis (GSEA) and over-representation analysis (ORA) were conducted on the indicated genes via ClusterProfiler packages [79, 80] (v.4.0.5). To perform gene ontology (GO) enrichment analysis, the “gseGO” and “enrichGO” functions were used, while the “gseKEGG” and “enrichKEGG” functions were utilized for Kyoto Encyclopedia of Genes and Genomes (KEGG) enrichment.

### Single-cell trajectory analysis

The Tools for Single Cell Analysis (TSCAN) tool [35] divides the single-cell data that have been clustered beforehand into several discrete clustering-based datasets, and computes the cluster centroids using the coordinates of the cells in each discrete cluster. The minimum spanning tree (MSN) was built on the basis of centroids of each cluster. Once the single-cell trajectory was constructed, Cells were going to be projected onto the trajectory and the distances of each cell from the root node (healthy NK cells were chosen as the root node of present trajectory) was computed, which is the pseudotime of that cell.

### Cell communication analysis

NK cell sub-categories based on cell death are integrated into the aggregate single-cell RNA sequencing data. CellChat [34] was performed on the aggregate scRNA seq to identify the cell communication. CellChat initially identifies the ligand or receptor genes overexpressed in each cell type, and then identifies the over-expressed receptor-ligand interactions among these overexpressed ligand and receptors. Subsequently, CellChat assigned probability values to every identified receptor-ligand interaction and performed permutation tests. According to the law of mass action, CellChat models the probability of cell-cell communication by integrating gene expression with prior known knowledge of the interactions between signaling ligands, receptors, and their cofactors. The number of inferred ligand-receptor pairs depends on the “trimean” average methods which performs well at predicting stronger interactions and is very helpful for narrowing down on interactions for further experimental validations. We integrated cell-cell communication by counting the number of cell interactions and calculating the probability of cell communication and then obtain the weight (strength) and number of receptor-ligand interaction on different cell types.

### Flow cytometry and cell sorting

Peripheral blood lymphocytes were extracted from the blood of trauma patients using density gradient centrifugation with human lymphocyte separation medium (TBD, #LTS1077). NK cells (CD3^−^CD56^+^) were identified by antibodies against CD3 (BD, #564713) and CD56 (BD, #562751). Expression of Annexin V and 7-AAD of NK cells was detected by BD Pharmingen™ PE Annexin V Apoptosis Detection Kit I (BD #559763). NK cell pyroptosis was labeled by GSDMD polyclonal antibody (Thermo Fisher, #PA5-119680) and Goat anti-Rabbit IgG (H + L) Cross-Adsorbed Secondary Antibody, Alexa Fluor™ 647 (Thermo Fisher, #A-21244). For the evaluation of NK cell autophagy, LC3 staining was carried out using the LC3-DyLight488 (Thermo Fisher, #PA5-22731). All fluorescent antibodies were detected by flow cytometry (BD, LSRFortessa^TM^) and analyzed the Flow Cytometry Standard data using FlowJo software (v10). Data visualization and statistical analysis is accomplished via GraphPad Prism (v9) and R (v4.1.0). Cell sorting was performed on FacsAriaIII cytometers (BD Biosciences). For experiments involving quantitative real-time PCR (qRT-PCR), cell populations were sorted to >95% purity. Data was analysis with FlowJo software.

### Single-cell RNA data acquisition

The scRNA traumatic cohort (GSE162806) was obtained from NCBI [20]. This cohort contained PBMCs scRNA information from 10 severe trauma patients at different time points and 10 age-, sex-matched healthy volunteers.

### Pseudo-bulk RNA generation

Initially, we identified the cell type and sample identifier for scRNA transcriptomes. Following this, we aggregated RNA expression per cell type based on sample identifier and obtained transcriptome information for each cell cluster, corresponding to sample-level resolution. The resolution of the aggregated single-cell datasets is consistent with that of bulk RNA transcriptomes. Therefore, we refer to this aggregated single-cell transcriptome as pseudo-bulk RNA.

### Weighted gene co-expression network analysis

The construction of gene co-expression networks was performed by the identification of differentially expressed genes (DEGs) from different paradigms of NK cells through the utilization of weighted gene co-expression network analysis (WGCNA) packages [81] (v3.3.4). The co-expression similarity matrix was constructed using Pearson correlations and transformed into an adjacency matrix through the application of soft-thresholding power (β), ensuring an appropriate fit for the scale-free topology and an extensive number of connections (the latter of which was achieved through the utilization of the “pickSoftThreshold” function). Gene networks were constructed with the condition *β* = 6 using the “blockwiseModules” function. Hypoxia-related modules were identified by detecting the correlation between different forms of NK cell death. The module eigengenes were determined using the “cor” functions, and the significance of the student p values was assessed using the “corPvalueStudent” function.

### Quantification and statical analysis

To compare two independent groups represented as bar plots, we conducted an unpaired two-tailed Student’s *t*-test with a 95% confidence interval in R (v4.1.0) to determine the *p* value. For data in violin plots, we performed a two-tailed Wilcoxon rank-sum test in R (v4.1.0). We determined the false discovery rate (FDR) using R (v4.1.0) to compare the differential gene expression analysis between bulk transcriptome and pseudo-bulk transcriptome RNA. Data are presented as the mean ± standard deviation if consistent with a normal distribution. A *p* value of less than 0.05 indicated statistically significant differences in the differential gene expression analysis. Differences were deemed significant when p < 0.05 (*), very significant when *p* < 0.01 (**), and highly significant when *p* < 0.001 (***).

### Quantitative real-time PCR

Cells were pre-enriched and then sorted to >95% purity and RNA was isolated using the RNeasy RNA purification mini (for 5 × 10^5^ cells or beyond). The complementary DNA (cDNA) was synthesized from purified RNA using the reverse-transcriptase qScript cDNA synthesis kit (Quanta Biosciences) to synthesize cDNA from purified RNA. qRT-PCR was conducted in triplicate on a 384-well plate using TaqMan probes (Thermo Fisher Scientific) or SYBR Green (Quanta Biosciences)-based detection on an ABI QuantStudio 6 Flex qRT-PCR machine. To analyzing mRNA abundance, the derived values were normalized according to GAPDH. Primer sequences are in Supplementary Table 3.

### Immunoblots

Sorted-purified NK cells (purity >95%) were collected directly into 20% trichloroacetic acid buffer from patients 4, 8 and 24 h post trauma. Following the sorting procedure, the concentration of trichloroacetic acid was adjusted to 10% and the mixture was incubated on ice for 30 min to facilitate the precipitation of proteins. The homogenates were subjected to centrifugation at 13,000 rpm. The samples were incubated at 4 °C for 10 min, after which supernatant was carefully removed. The pellets with concentrated proteins were washed twice with acetone and then solubilized in solubilization buffer (containing 9 M urea, 2% Triton X-100 and 1% dithiothreitol) mixed with LDS buffer (Invitrogen). The samples were subjected to a 10 min heating process at 70 °C, after which the protein concentrations were determined through the use of a BCA protein assay kit (Thermo Fisher Scientific). Equivalent amounts of protein for NK cells were separated by membranes following the standard protocol. Anti-LC3 (14600-1-AP, Proteintech), anti-β-Actin (6609-1-1g, Proteintech) and HRP-Coat anti-rabbit recombination secondary antibody (RGAR001, Proteintech) were used. SuperKine™ West Femto Maximum Sensitivity Substrate (BMU102, Abbkine) was used to image blots in a ChemiDoc MP Imaging System instrument (BioRad).

### Supplementary information

Supplementary Information

Original Data File

## Data Availability

The raw data supporting the conclusions of this article will be made available from the corresponding author on reasonable request. The datasets presented in this study can be found in online repositories. The names of the repository/repositories and accession number(s) can be found below: https://www.ncbi.nlm.nih.gov/, GSE197522.

## Code availability

The protocol and data analysis were obtained from the official websites of the 10X Genome and Seurat. The functions and their parameters are detailed in the corresponding sections of “Method”. Upon reasonable request, we can provide custom scripts for data analysis. Source data are provided with this article.
