# Supplementary material for: The different paradigms of NK cell death in patients with severe trauma
Source: Cell Death Dis. 2024 Aug 21;15(8):606. doi: 10.1038/s41419-024-06992-0 (PMC11339281; doi:10.1038/s41419-024-06992-0)
Supplement: Supplementary file 1 — Supplementary Information [file 41419_2024_6992_MOESM1_ESM.docx]

**SUPPLEMENTARY INFORMATON**

**The different paradigms of NK cell death in patients with severe trauma**

Te-Ding Chang ^1,2^, Deng Chen ^1,2^, Jia-Liu Luo ^1,2^, Yu-Man Wang ^3^, Cong Zhang ^1,2^, Shun-Yao Chen ^1,2^, Zhi-Qiang Lin ^1,2^, Pei-Dong Zhang ^1,2^, Ting-Xuan Tang ^5^, Hui Li ^1,2^, Li-Ming Dong ^1,2^, Ning Wu ^3,4^, Zhao-Hui Tang ^1,2,^^§^

^1^ Division of Trauma Surgery, Emergency Surgery & Surgical Critical, Tongji Trauma Center, Tongji Hospital, Tongji Medical College, Huazhong University of Science and Technology, Wuhan, China.

^2^ Department of Emergency and Critical Care Medicine, Tongji Hospital, Tongji Medical College, Huazhong University of Science and Technology, Wuhan, China.

^3^ Department of Immunology, School of Basic Medicine, Tongji Medical College, Huazhong University of Science and Technology, Wuhan, China

^4^ Department of Immunology, School of Basic Medical Sciences, Anhui Medical University, Hefei, China

^5^ Department of Orthopedics, Tongji Hospital, Tongji Medical College, Huazhong University of Science and Technology, Wuhan, China

^§^ Correspondence: [tangzh@tjh.tjmu.edu.cn](mailto:tangzh@tjh.tjmu.edu.cn) (Zhao-Hui Tang)

**Supplementary Figures**


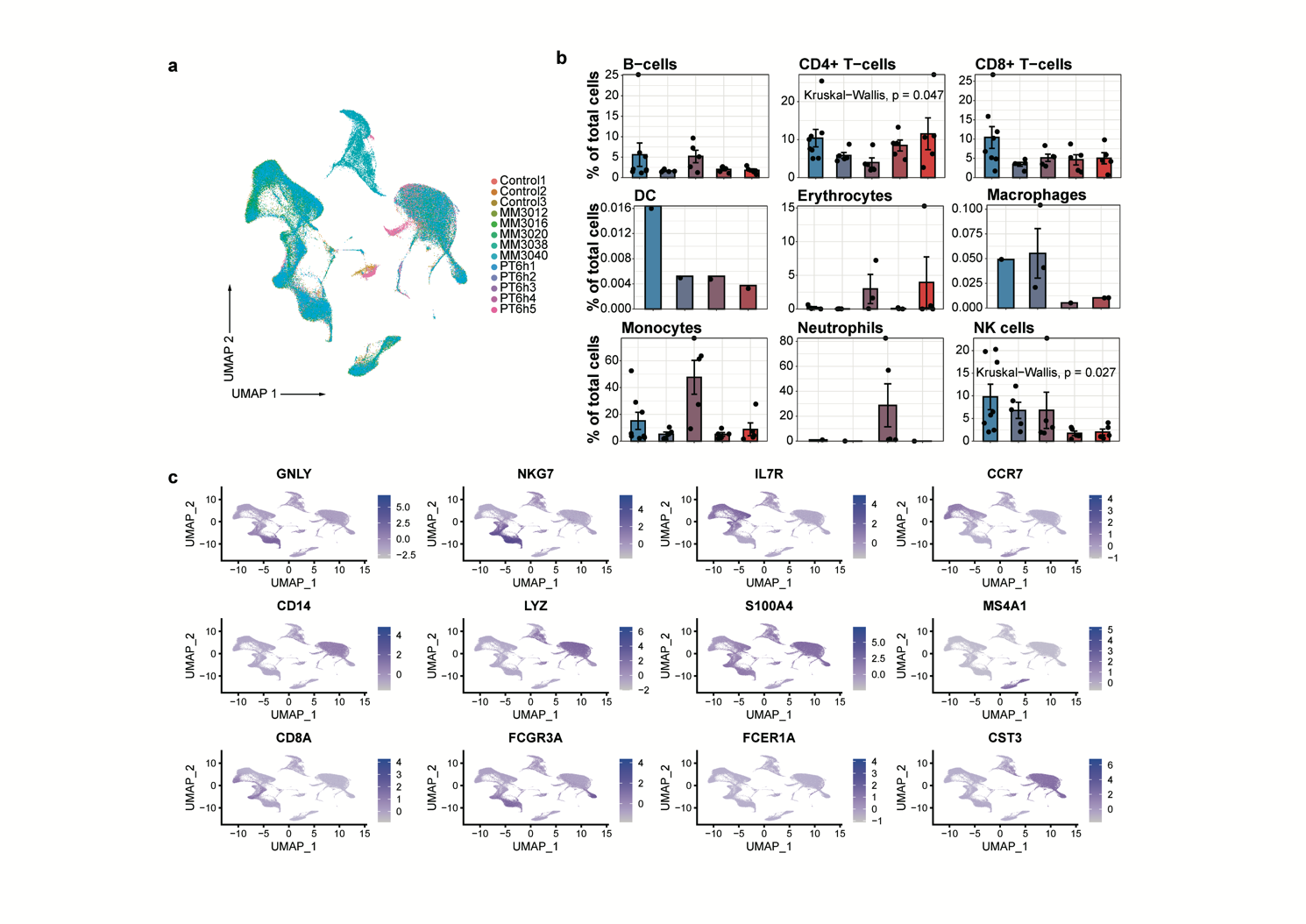


**Supplementary Figure 1.** **scRNA-seq identifies trauma-specific immune-cell states and gene signatures.** **a.** UMAP plot of trauma-induced PBMCs after mutual nearest neighbor (MNN) correction. Each point represents a cell that is colored according to its batch of origin. Control 1-3 and PT6h 1-5 come from our traumatic cohorts. MM3012, MM3016, MM3020, MM3048, and MM3040 were obtained from the traumatic database GSE162806. **b.** Fraction of total trauma-induced immune cells across each post-trauma time point. FDR values are shown when comparing each time point with healthy controls (Mean ± SEM, two-tailed Wilcoxon rank-sum test, corrected for testing of multiple states). **c.** Expression of select marker genes across PBMCs as visualized on UMAP plot. Each point represents a cell and is colored by the marker gene expression levels.


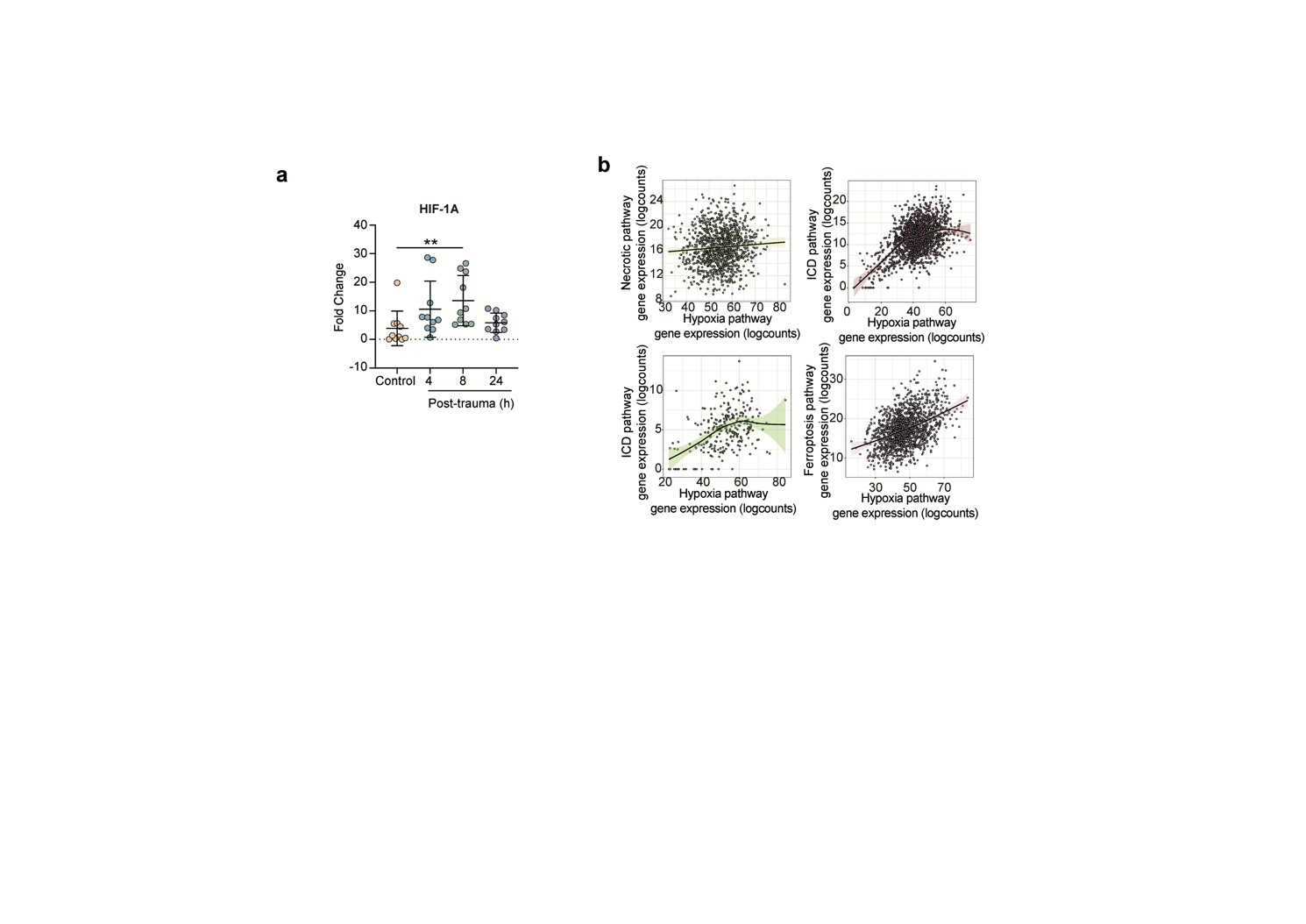


**Supplementary Figure 2.** **Correlation between hypoxia-related pathways and cell death. a,** qRT-PCR analysis of HIF-1A from purified NK cells of isolated cohort of trauma patients (n=30, PT4h, PT6h and PT24h). Error bars represent mean with s.d. the *p* value was calculated by unpaired, two-tail Student’s t-test. ****p* < 0.001, ***p* < 0.01, and **p* < 0.05. **b,** The correlation between hypoxia and Necrotic, ICD, autophagy, ferroptosis pathway genes in NK cells. The fitted curves were obtained using loess regression. Shaded areas show 95% confidence intervals for the fitted curves. P value was calculated by one-way ANOVA, ****p* < 0.001, ***p* < 0.01, and **p* < 0.05.


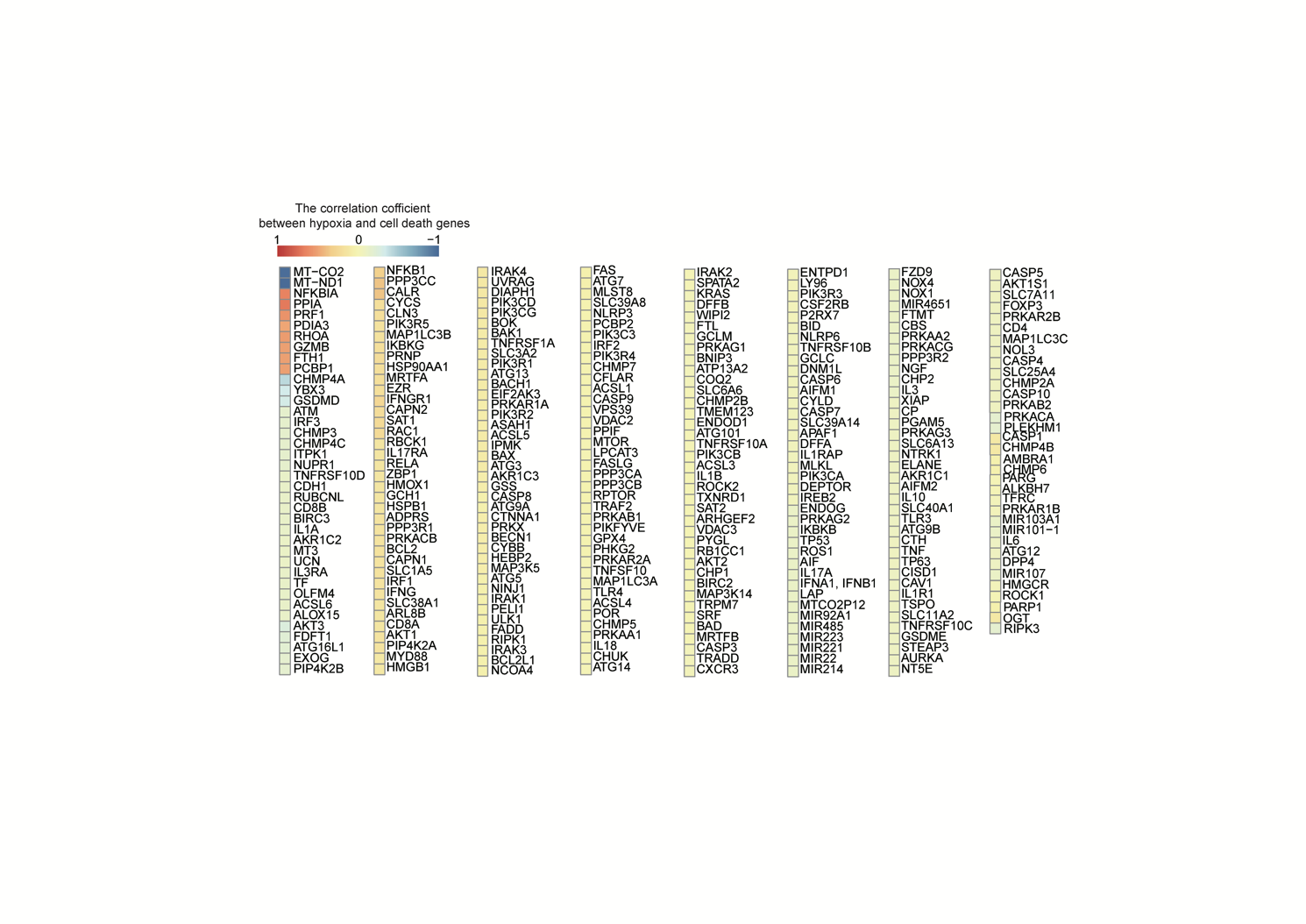


**Supplementary Figure 3.** **Correlation of hypoxia with cell death genes.** The heat map of spearman correlation between hypoxia and all cell death genes, with colors representing the correlation coefficient between hypoxia and cell death genes.


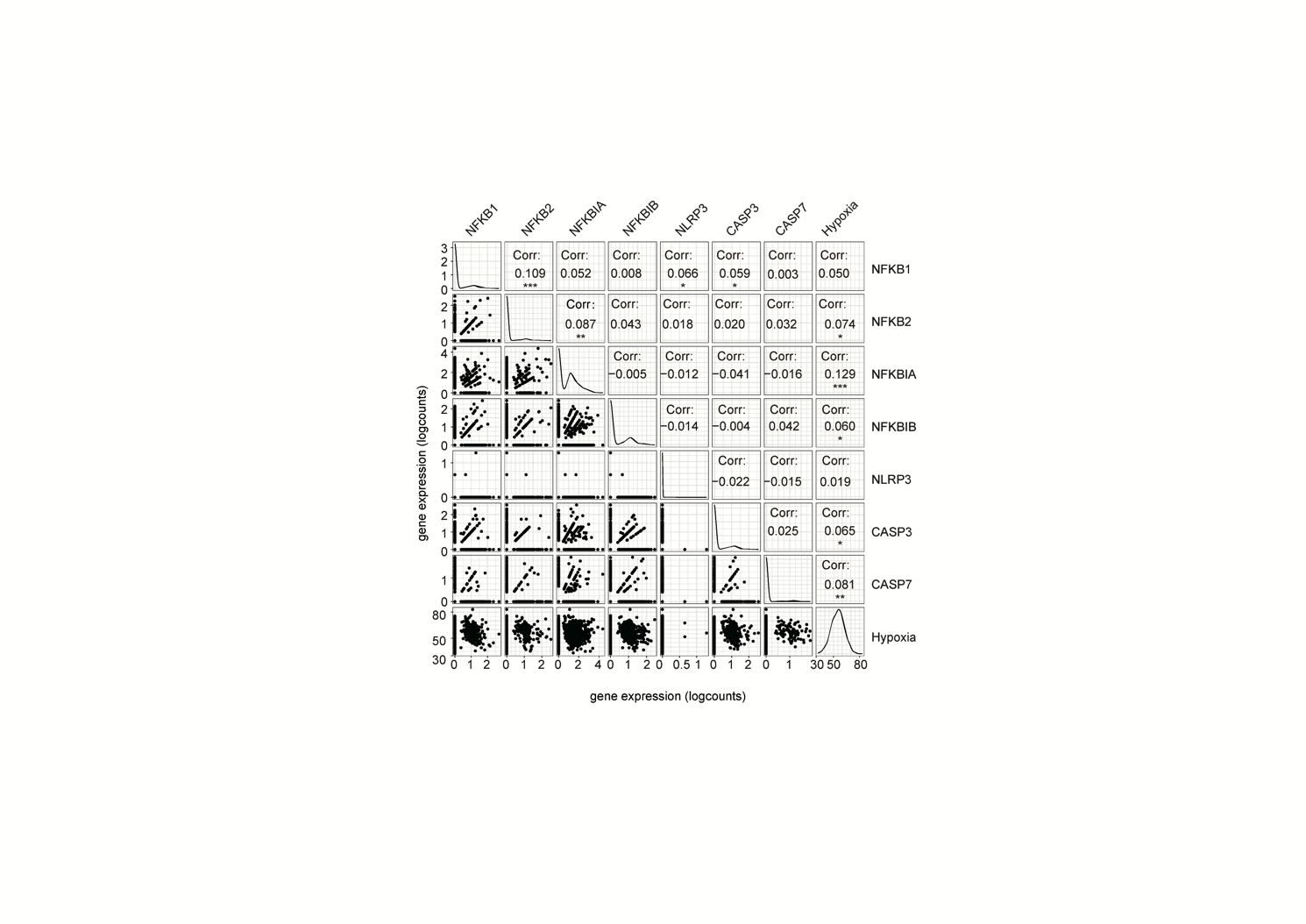


**Supplementary Figure 4.** **Correlation of critical genes of pyroptosis and apoptosis with hypoxia.** Top triangular region shows the Pearson correlation and the p-value. Diagonal line shows the distribution curve of different genes. Bottom triangular region shows the scatter plots of the two genes. the *p* values were calculated by one-way ANOVA, ****p* < 0.001, ***p* < 0.01, and **p* < 0.05.


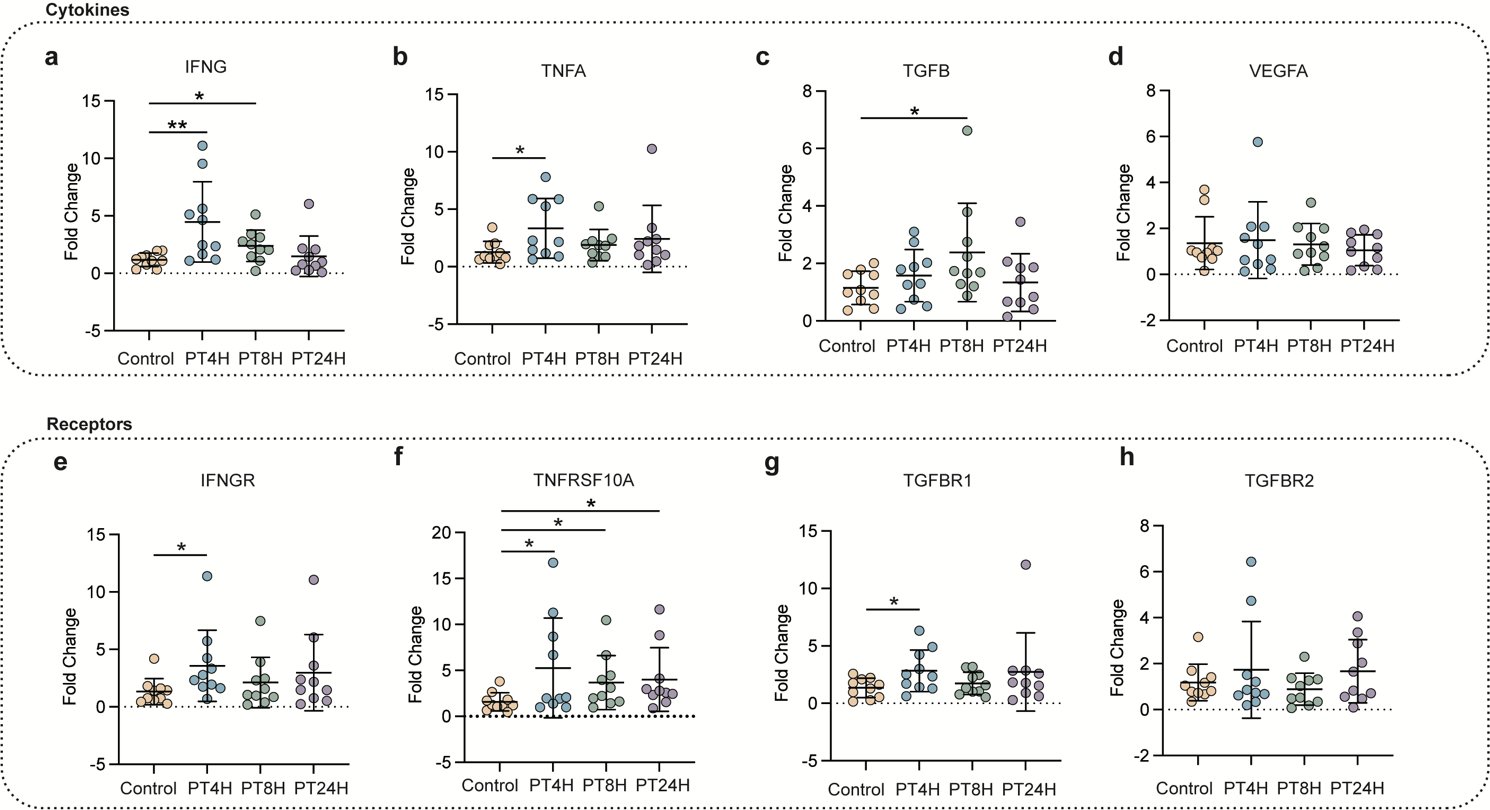


**Supplementary Figure 5.** **Validation qRT-PCR analysis of critical cytokines and receptors from purified NK cells from validation cohort of trauma patients.** **a-d,** qRT-PCR analysis of IFNγ (**a**), TNFα (**b**), TGFβ (**c**) and VEGFA (**d**) from purified NK cells of trauma patients in the isolated cohort. **e-f,** qRT-PCR analysis of IFNGR(**e**), TNFRSF10A (**f**), TGFBR1 (**g**) and TGFBR2 (**h**) from purified NK cells of trauma patients in the isolated cohort. All error bars represent mean with s.d. the *p* values were calculated by unpaired, two-tail Student’s t-test. ****p* < 0.001, ***p* < 0.01, and **p* < 0.05.


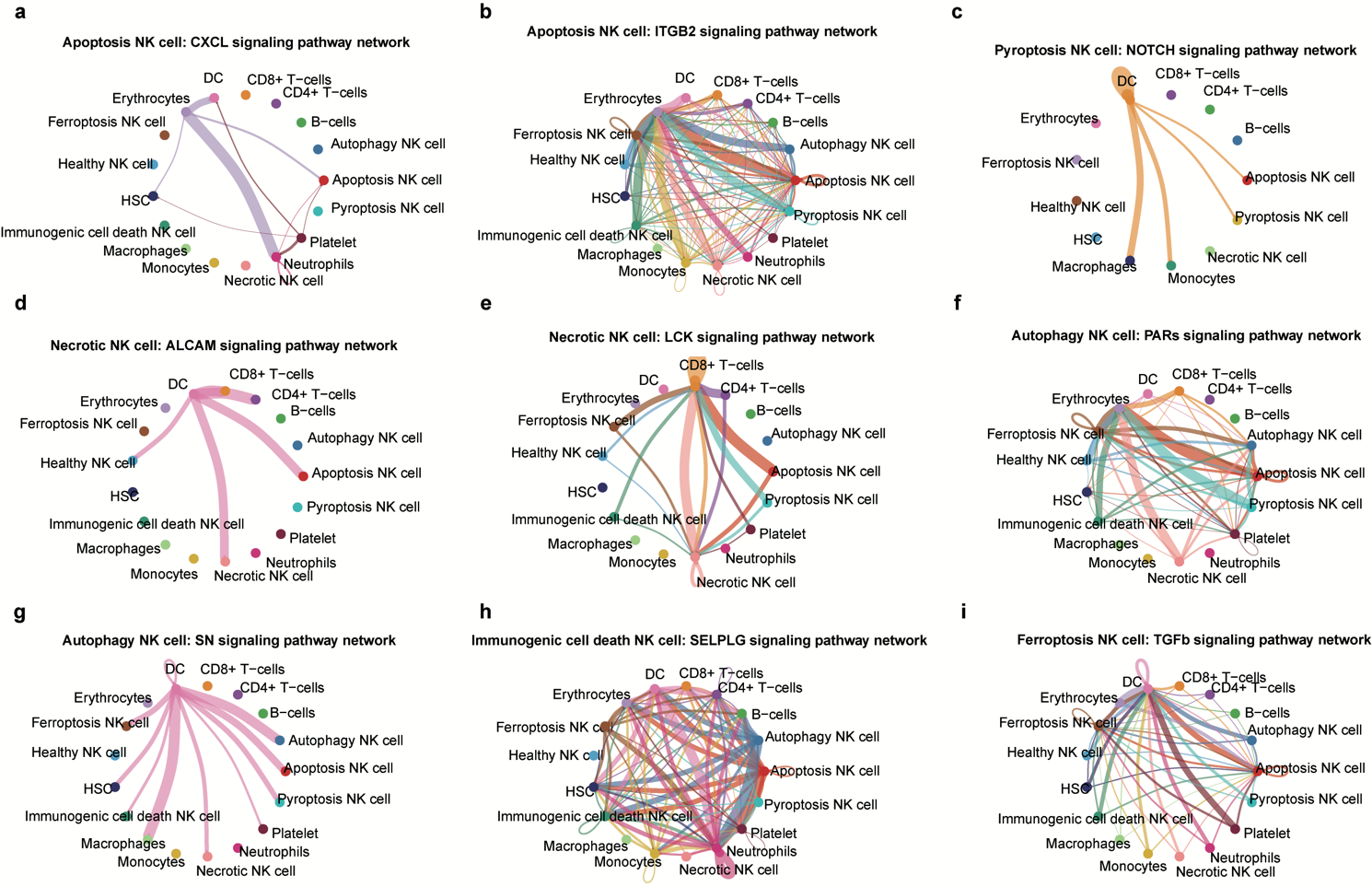


**Supplementary Figure 6.** **Network diagram of cell communication between NK cells and other immune cells.** **a-i.** Network diagram of different signaling pathways for different death patterns of NK cells communicating with other immune cells. Colors of the line represent different immune cells, and thicknesses represent weight value for communicating.

**Supplementary Tables**

**Supplementary table 1: Patient characteristics in validated cohort**

| **Post Trauma, hours** | **4 hours (n = 10)** | **8 hours (n = 10)** | **24 hours (n = 10)** |
| --- | --- | --- | --- |
| Age, median (IQR), y | 50 (46-55.25) | 46.5 (46-55.25) | 46 (46-52.5) |
| Sex, No. (%) | | | |
| Female | 40% | 40% | 50% |
| Male | 60% | 60% | 50% |
| Mechanism of injury, No. (%) | | | |
| Traffic accidents | 50% | 40% | 60% |
| Falls | 40% | 40% | 30% |
| Crushes | 10% | 20% | 10% |
| GCS, median (IQR) | 10 (7-12) | 8.5 (6.25-10) | 10.5 (9.25-12.5) |
| ISS, median (IQR) | 28 (23-32.75) | 28 (22-29) | 26 (22-28.25) |
| **Abbreviations:**  IQR: inter-quartile range.  GCS: Glasgow Coma Scale.  ISS: Injury severity score. | | | |

**Supplementary table 2: Cell death genes**

| **Pathways** | **Genes** | **Database** |
| --- | --- | --- |
| apoptosis | AIFM1;AKT1;AKT2;AKT3;APAF1;ATM;BAD;BAX;BCL2;BCL2L1;BID;BIRC2;BIRC3;CAPN1;CAPN2;CASP10;CASP3;CASP6;CASP7;CASP8;CASP9;CFLAR;CHP1;CHP2;CHUK;CSF2RB;CYCS;DFFA;DFFB;ENDOD1;ENDOG;EXOG;FADD;FAS;FASLG;IKBKB;IKBKG;IL1A;IL1B;IL1R1;IL1RAP;IL3;IL3RA;IRAK1;IRAK2;IRAK3;IRAK4;MAP3K14;MYD88;NFKB1;NFKBIA;NGF;NTRK1;PIK3CA;PIK3CB;PIK3CD;PIK3CG;PIK3R1;PIK3R2;PIK3R3;PIK3R5;PPP3CA;PPP3CB;PPP3CC;PPP3R1;PPP3R2;PRKACA;PRKACB;PRKACG;PRKAR1A;PRKAR1B;PRKAR2A;PRKAR2B;PRKX;RELA;RIPK1;TNF;TNFRSF10A;TNFRSF10B;TNFRSF10C;TNFRSF10D;TNFRSF1A;TNFSF10;TP53;TRADD;TRAF2;XIAP | KEGG |
| pyroptosis | BAK1;BAX;CASP1;CASP3;CASP4;CASP5;CHMP2A;CHMP2B;CHMP3;CHMP4A;CHMP4B;CHMP4C;CHMP6;CHMP7;CYCS;ELANE;GSDMD;GSDME;GZMB;HMGB1;IL18;IL1A;IL1B;IRF1;IRF2;TP53;TP63 | REACTOME |
| necrotic cell death | DPRS; ALKBH7; ARHGEF2; ASAH1; ATG9A; ATG9B; BAX; BIRC2; BIRC3; BNIP3; BOK; CASP6; CASP8; CAV1; CFLAR; CYLD; DNM1L; FADD; FAS; FASLG; FZD9; GSDME; HEBP2; IPMK; IRF3; ITPK1; MAP3K5; MIR101-1; MIR103A1; MIR107; MIR214; MIR22; MIR221; MIR223; MIR485; MIR92A1; MLKL; MT-CO2; MT3; MTCO2P12; NINJ1; NLRP6; NOL3; NUPR1; OGT; OLFM4; PELI1; PGAM5; PPIF; PYGL; RBCK1; RIPK1; RIPK3; SLC25A4; SLC6A13; SLC6A6; SPATA2; TLR3; TMEM123; TNF; TP53; TRAF2; TRPM7; TSPO; UCN; YBX3; ZBP1 | GOBP |
| Necroptotic | FADD; FAS; FASLG; MLKL; RIPK1; RIPK3; TLR3; TNF | GOBP |
| Immunological cell death | ENTPD1; NT5E; CALR; HMGB1; HSP90AA1; ATG5; BAX; CASP8; PDIA3; EIF2AK3; PIK3CA; CXCR3; IFNA1, IFNB1; IL10; IL6; TNF; CASP1; IL1R1; IL1B; NLRP3; P2RX7; LY96; MYD88; TLR4; CD4; CD8A; FOXP3; CD8B; IFNG; IFNGR1; IL17A; IL17RA; PRF1 | Nomenclature Committee on Cell Death 2018 |
| Ferroptosis | ACSL1; ACSL3; ACSL4; ACSL5; ACSL6; AIFM2; AKR1C1; AKR1C2; AKR1C3; ALOX15; ATG5; ATG7; BACH1; CBS; CHMP5; CHMP6; CISD1; COQ2; CP; CTH; CYBB; DPP4; FDFT1; FTH1; FTL; FTMT; GCH1; GCLC; GCLM; GPX4; GSS; HMGCR; HMOX1; HSPB1; IREB2; LPCAT3; MAP1LC3A; MAP1LC3B; MAP1LC3C; MIR4651; NCOA4; NOX1; NOX4; PCBP1; PCBP2; PHKG2; POR; PRNP; SAT1; SAT2; SLC11A2; SLC1A5; SLC38A1; SLC39A14; SLC39A8; SLC3A2; SLC40A1; SLC7A11; STEAP3; TF; TFRC; TP53; TXNRD1; VDAC2; VDAC3 | WikiPathways |
| Autophagy | AKT1S1; AMBRA1; ATG101; ATG12; ATG13; ATG14; ATG16L1; ATG3; ATG5; ATG7; ATG9A; BECN1; DEPTOR; MAP1LC3B; MLST8; MTOR; PIK3C3; PIK3R4; PRKAA1; PRKAA2; PRKAB1; PRKAB2; PRKAG1; PRKAG2; PRKAG3; RB1CC1; RPTOR; ULK1; UVRAG; WIPI2 | WikiPathways |
| Autophagosome lysosome fusion | ARL8B; ATP13A2; CLN3; PIP4K2A; PIP4K2B; PLEKHM1; RUBCNL; VPS39 | GOBP |
| Parthanatos | AIF; ADPRS; PPIA; ENDOG; AIFM1; PARP1; PARG; MT-ND1; ROS1 | Nomenclature Committee on Cell Death 2018 |
| Entotic cell death | CDH1; CTNNA1; RHOA; ROCK1; ROCK2; DIAPH1; MRTFA; MRTFB; SRF; EZR; AURKA; KRAS; RAC1; PRKAA1; LAP; MAP1LC3B; ATG5; ATG7; PIK3C3; PIKFYVE | Nomenclature Committee on Cell Death 2018 |

# **Supplementary Table 3: Primers used for qRT-PCR**

| **Species** | **Gene Target** | **Forward Primer**  **(5’-3’)** | **Reverse Primer**  **(5’-3’)** |
| --- | --- | --- | --- |
| Homo sapiens | HIF1A | TATGAGCCAGAAGAACTTTTAGGC | CACCTCTTTTGGCAAGCATCCTG |
| Homo sapiens | IFNG | GAGTGTGGAGACCATCAAGGAAG | TGCTTTGCGTTGGACATTCAAGTC |
| Homo sapiens | TNFA | CTCTTCTGCCTGCTGCACTTTG | ATGGGCTACAGGCTTGTCACTC |
| Homo sapiens | TGFB | TACCTGAACCCGTGTTGCTCTC | GTTGCTGAGGTATCGCCAGGAA |
| Homo sapiens | VEGFA | TTGCCTTGCTGCTCTACCTCCA | GATGGCAGTAGCTGCGCTGATA |
| Homo sapiens | IFNGR1 | AGTGCTTAGCCTGGTATTCATCTG | GGCTGGTATGACGTGATGAGTG |
| Homo sapiens | TNFRSF10A | GTGTGGGTTACACCAATGCTTCC | CCTGGTTTGCACTGACATGCTG |
| Homo sapiens | TGFBR1 | GACAACGTCAGGTTCTGGCTCA | CCGCCACTTTCCTCTCCAAACT |
| Homo sapiens | TGFBR2 | GTCTGTGGATGACCTGGCTAAC | GACATCGGTCTGCTTGAAGGAC |
| Homo sapiens | GAPDH | GTCTCCTCTGACTTCAACAGCG | ACCACCCTGTTGCTGTAGCCAA |
