# Supplementary figures and images for: The different paradigms of NK cell death in patients with severe trauma
Source: Cell Death Dis. 2024 Aug 21;15(8):606. doi: 10.1038/s41419-024-06992-0 (PMC11339281; doi:10.1038/s41419-024-06992-0)

Figure 1h

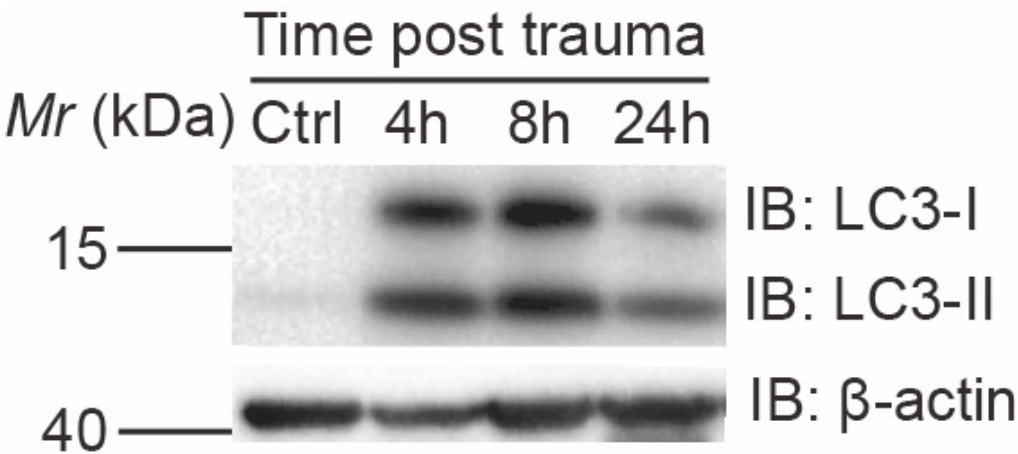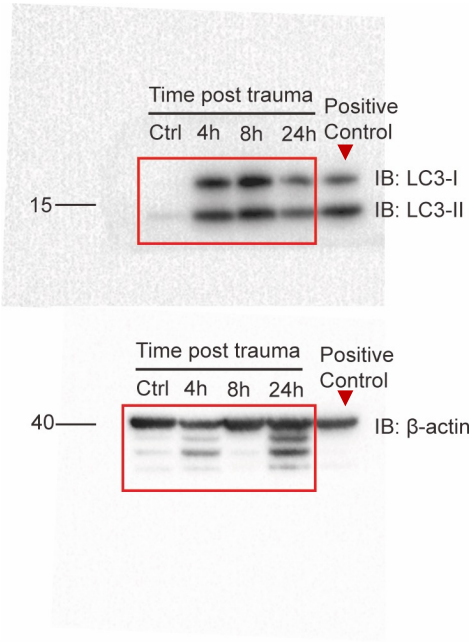

Supplement: Supplementary file 2 — Original Data File [file 41419_2024_6992_MOESM2_ESM.pdf]
